# Supplementary material for: Shallow Whole-Genome Sequencing from Plasma Identifies FGFR1 Amplified Breast Cancers and Predicts Overall Survival
Source: Cancers (Basel). 2020 Jun 6;12(6):1481. doi: 10.3390/cancers12061481 (PMC7353062; doi:10.3390/cancers12061481)
Supplement: Supplementary file 1 [file cancers-12-01481-s001.pdf]

Supplementary Materials: Shallow whole-genome sequencing from plasma identifies FGFR1 amplified breast cancers and predicts overall survival

Chantal Bourrier, Jean-Yves Pierga, Laura Xuereb, Hélène Salaun, Charlotte Proudhon, Michael Speicher, Jelena Belic, Ellen Heitzer, Brian Paul Lockhart and Nolwen Guigal-Stephan

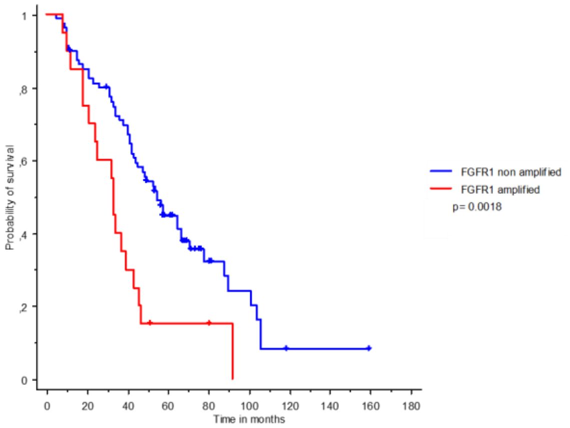

Figure S1. Overall Survival according to FGFR1 Amplification.

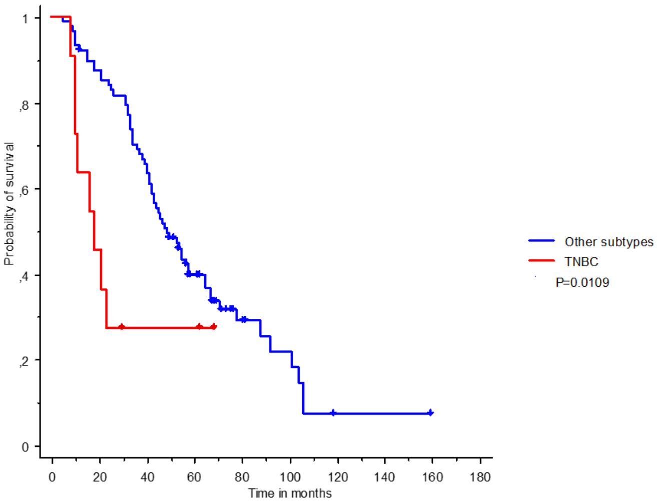

Figure S2. overall survival according to histological subtype (Log Rank).

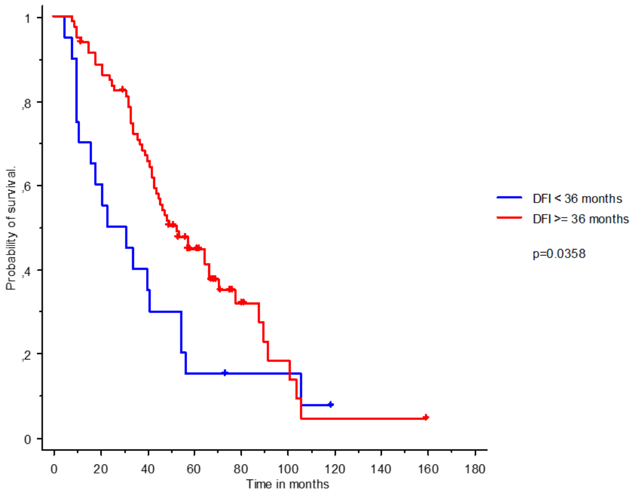

Figure S3. Overall survival according to Disease Free Interval in months (Log Rank).

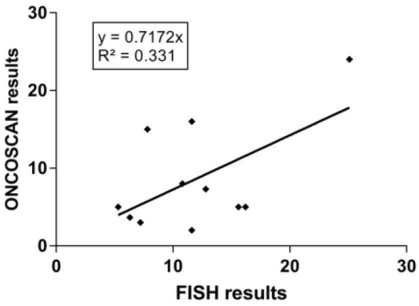

Figure S4. Correlation between Oncoscan and FISH results.

FGFR1 amplification positive samples & detected in plasma DNA

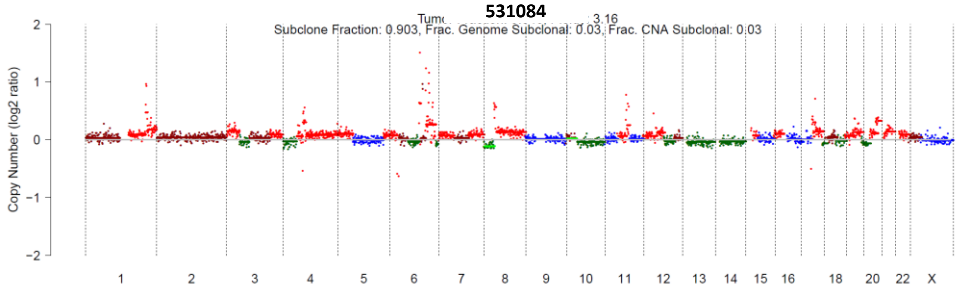

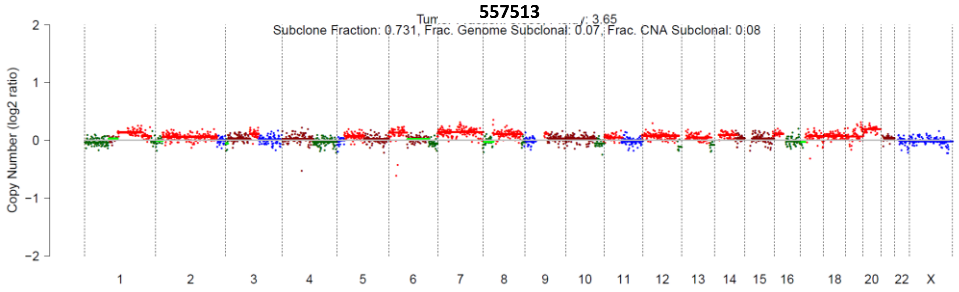

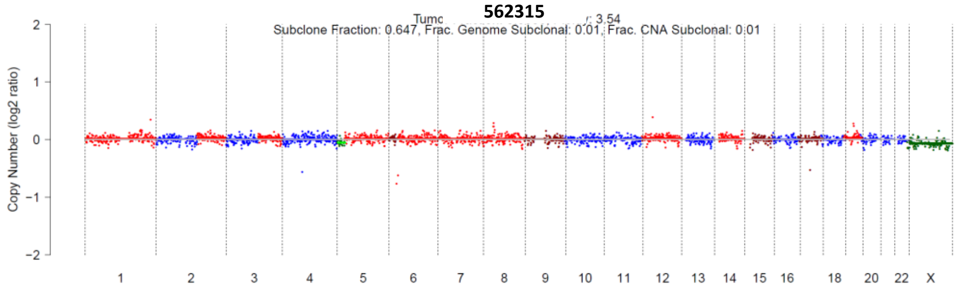

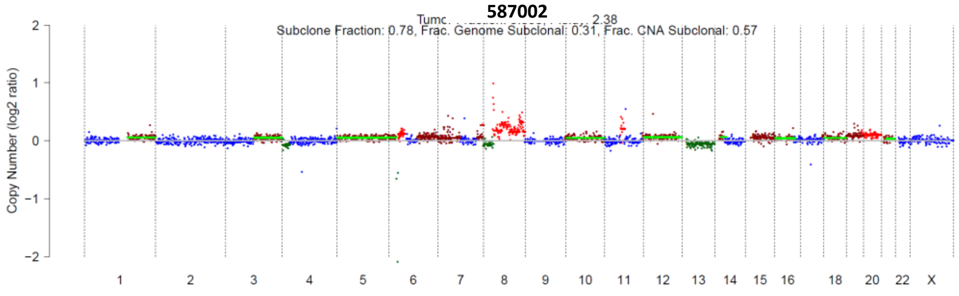

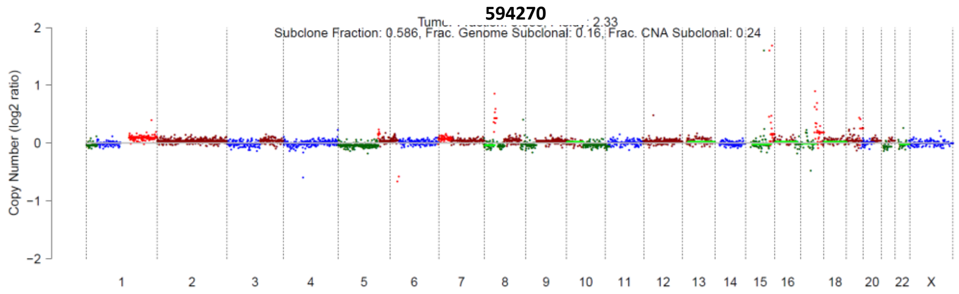
594270
Subclone Fraction: 0.586, Frac. Genome Subclonal: 0.16, Frac. CNA Subclonal: 0.24
Copy Number (log2 ratio)
1 2 3 4 5 6 7 8 9 10 11 12 13 14 15 16 18 20 22 X

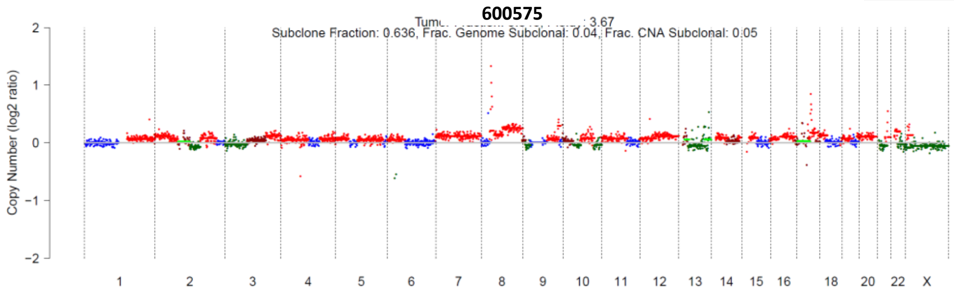
600575
Subclone Fraction: 0.636, Frac. Genome Subclonal: 0.04, Frac. CNA Subclonal: 0.05
Copy Number (log2 ratio)
1 2 3 4 5 6 7 8 9 10 11 12 13 14 15 16 18 20 22 X

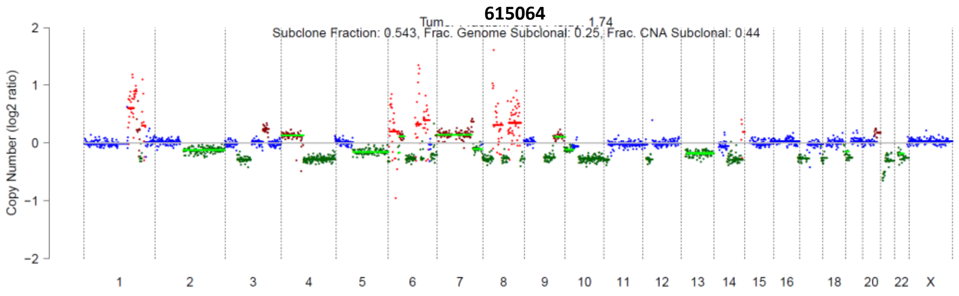
615064
Subclone Fraction: 0.543, Frac. Genome Subclonal: 0.25, Frac. CNA Subclonal: 0.44
Copy Number (log2 ratio)
1 2 3 4 5 6 7 8 9 10 11 12 13 14 15 16 18 20 22 X

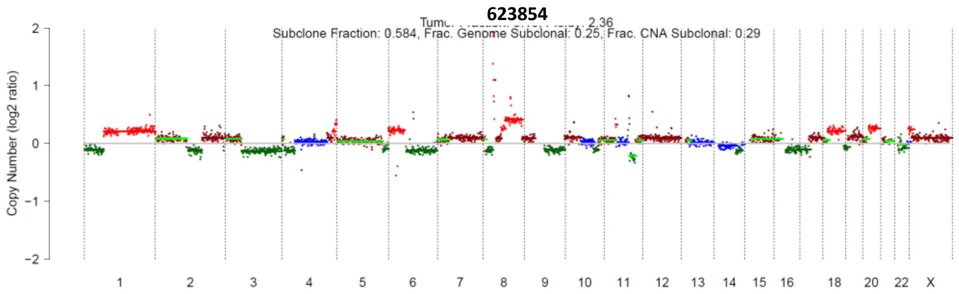
623854
Subclone Fraction: 0.584, Frac. Genome Subclonal: 0.25, Frac. CNA Subclonal: 0.29
Copy Number (log2 ratio)
1 2 3 4 5 6 7 8 9 10 11 12 13 14 15 16 18 20 22 X

FGFR1 amplification positive samples & NOT detected in plasma DNA

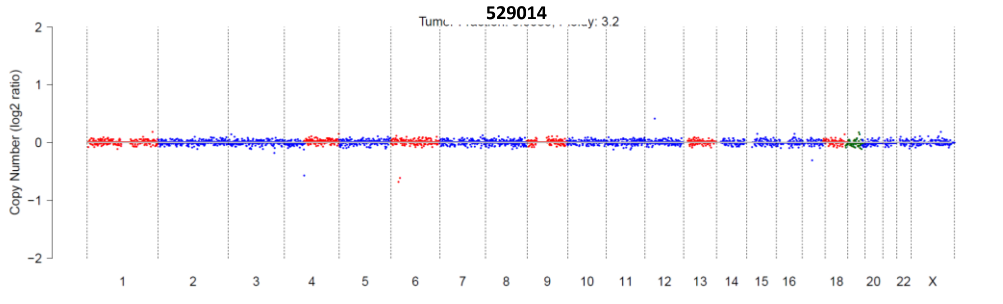

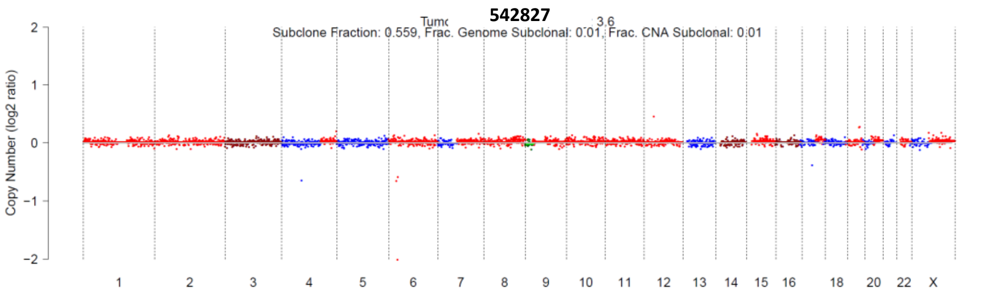

FGFR1 amplification negative samples

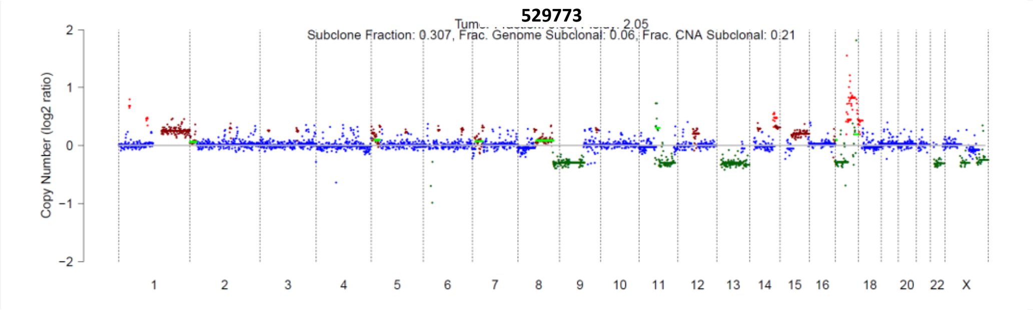

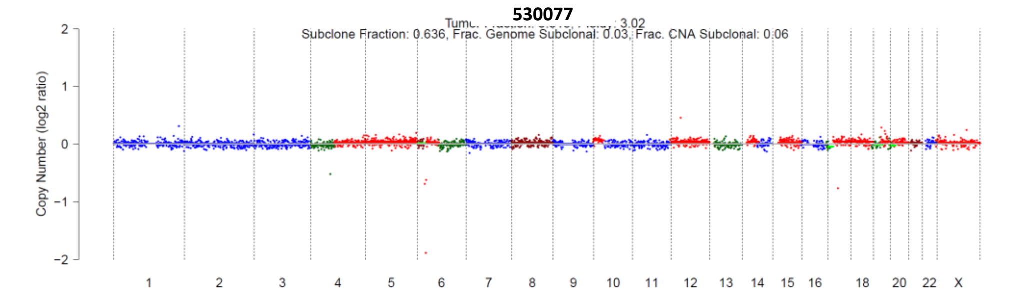

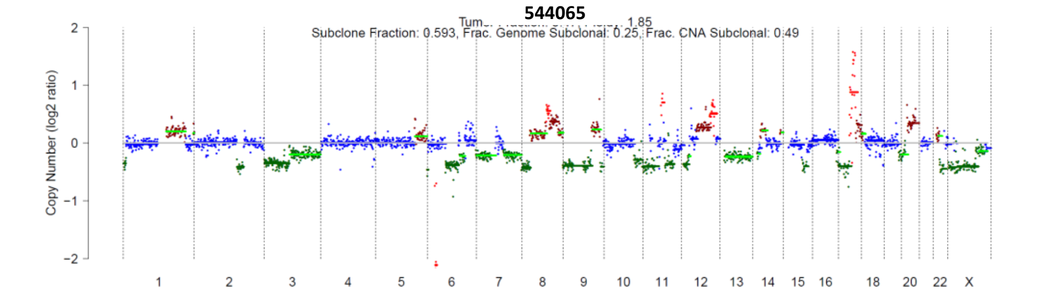

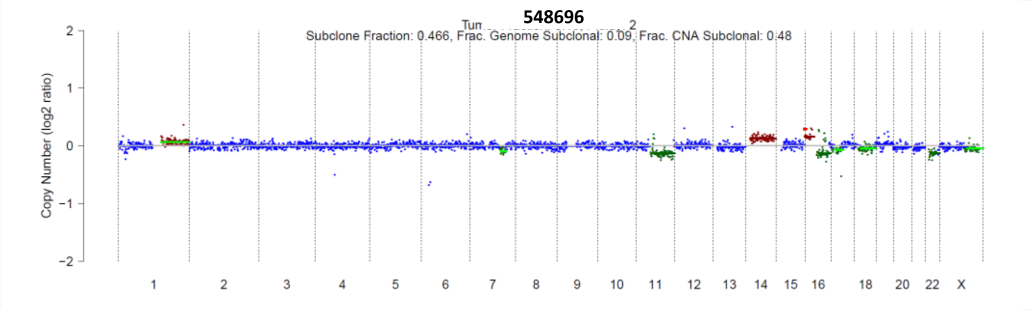

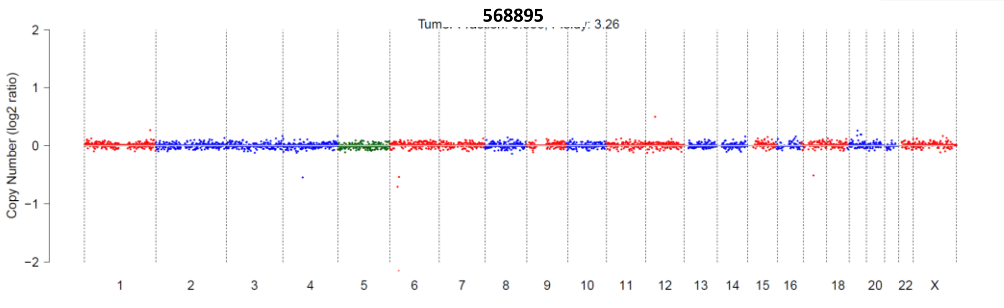
568895
Copy Number (log2 ratio)
1 2 3 4 5 6 7 8 9 10 11 12 13 14 15 16 18 20 22 X

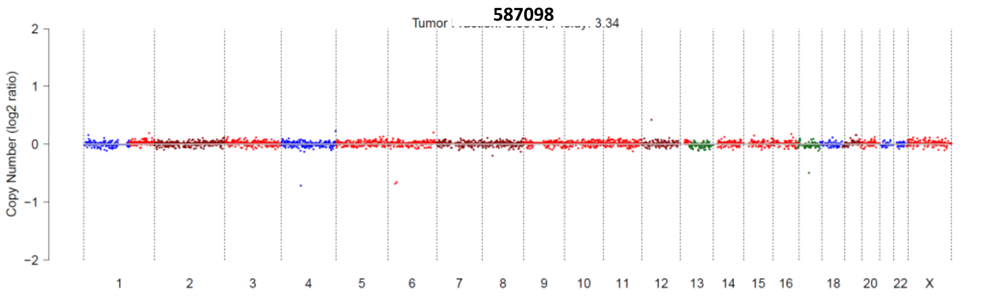
587098
Copy Number (log2 ratio)
1 2 3 4 5 6 7 8 9 10 11 12 13 14 15 16 18 20 22 X

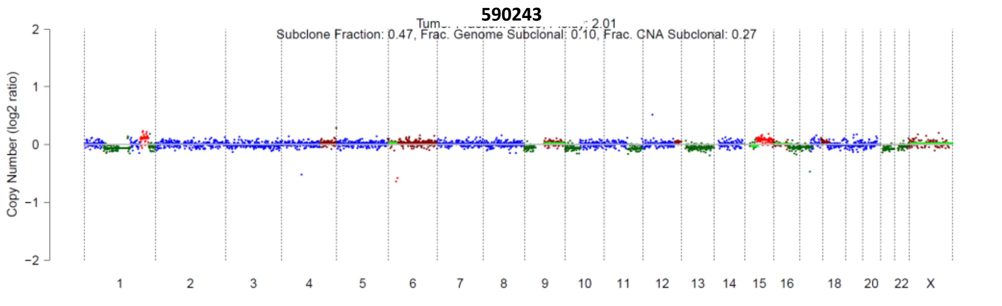
590243
Subclone Fraction: 0.47, Frac. Genome Subclonal: 0.10, Frac. CNA Subclonal: 0.27
Copy Number (log2 ratio)
1 2 3 4 5 6 7 8 9 10 11 12 13 14 15 16 18 20 22 X

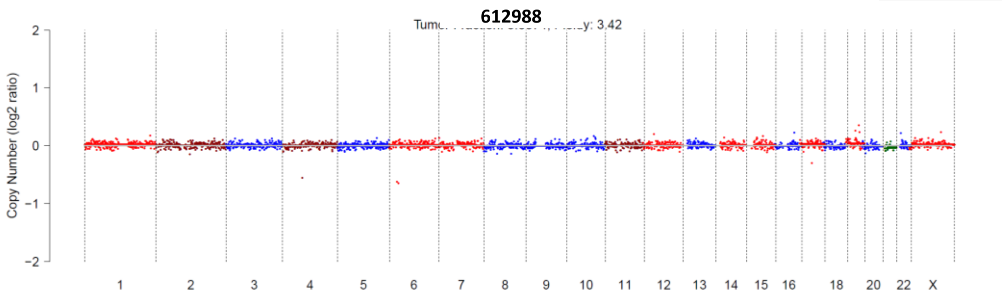
612988
Copy Number (log2 ratio)
1 2 3 4 5 6 7 8 9 10 11 12 13 14 15 16 18 20 22 X

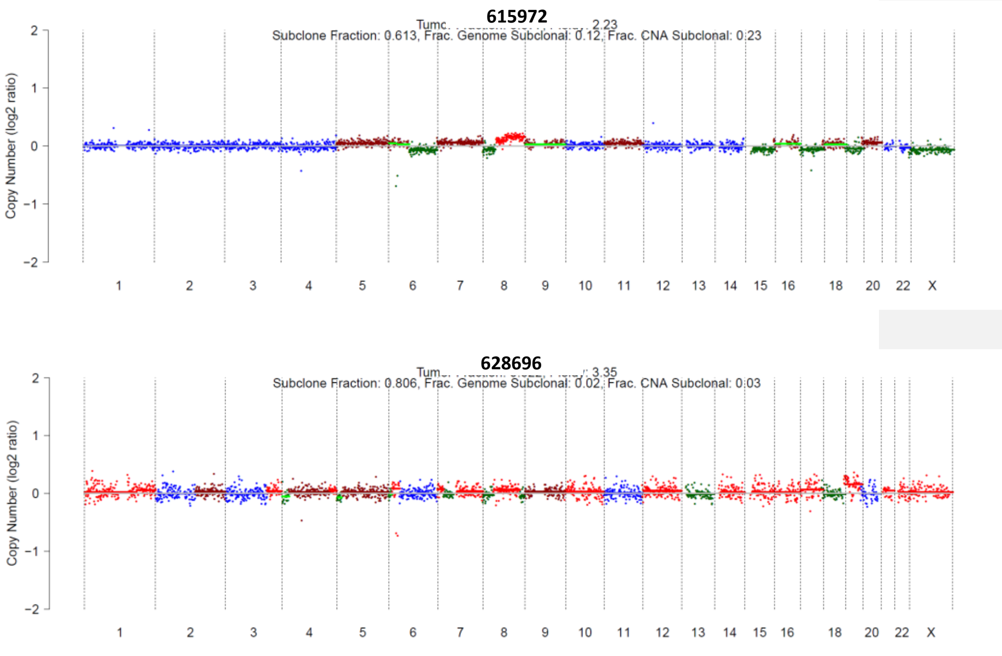

Figure S5: Detailed copy number profiles for each patient sample obtained by sWGS

Commented [BCI1]: Figure 5 was previously sent in another file. We added figures in this document.

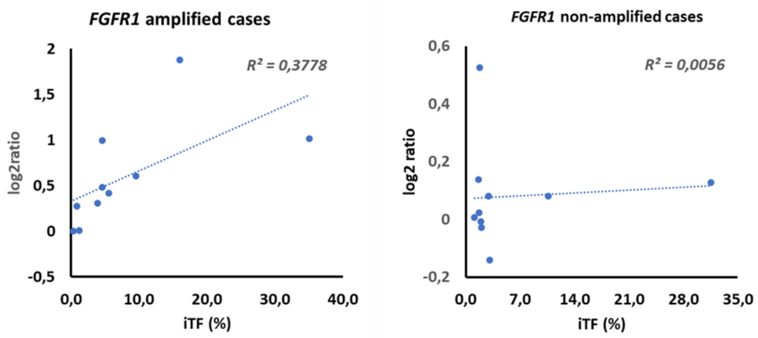

Figure S6. Correlation of tumour fractions FGFR1 log2ratios in plasma. Shown is a linear regression of the ichorCNA-derived tumour fraction (iTF) and respective log2ratio of a 1Mb genomic region harbouring the FGFR1 gene. Left plot, plasma DNA data from FGFR1 amplified cases. Right plot, plasma DNA data from FGFR1 non-amplified cases.

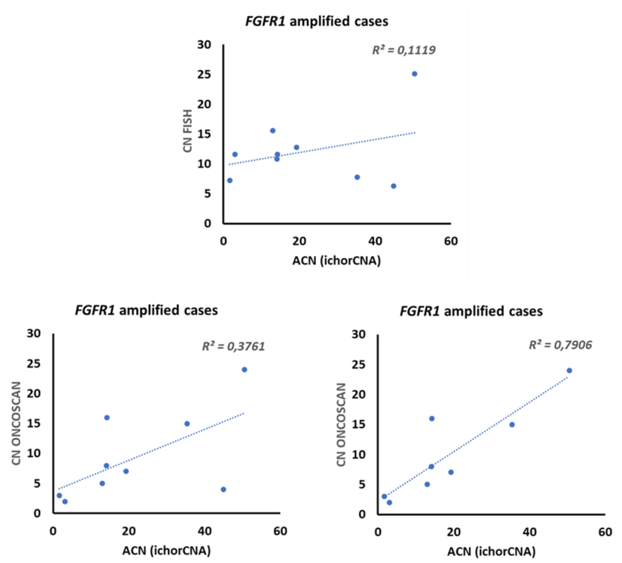

Figure S7. Correlation of copy numbers from three orthogonal approaches. Shown is a linear regressions of the copy number (CN) established from FISH, ONCOSCAN and sWGS. Hypothetical absolute copy numbers (ACN) were calculated form the ichorCNA-derived tumour fraction (iTF) and respective log2ratio for FGFR1. Upper plot, Correlation of FISH and ACN from ichorCNA. Lower left plot, Correlation of ONCOSCAN and ACN from ichorCNA. Lower right plot, Correlation of ONCOSCAN and ACN from ichorCNA without one outlier.

© 2020 by the authors. Submitted for possible open access publication under the terms and conditions of the Creative Commons Attribution (CC BY) license (http://creativecommons.org/licenses/by/4.0/).
